# Molecular treatment trajectories within psoriatic T lymphocytes: a mini review

**DOI:** 10.3389/fimmu.2023.1170273

**Published:** 2023-05-12

**Authors:** Martyna Kuczyńska, Magdalena Gabig-Cimińska, Marta Moskot

**Affiliations:** Department of Medical Biology and Genetics, University of Gdańsk, Gdańsk, Poland

**Keywords:** autoimmunity, autoinflammation, psoriasis, skin-resident and circulating T lymphocytes, molecular treatment trajectories, small molecule drugs (SMDs)

## Abstract

Multiple biological processes in mammalian cells are implicated in psoriasis (Ps) development and progression, as well as in the pathogenic mechanisms associated with this chronic immune-mediated inflammatory disease (IMID). These refer to molecular cascades contributing to the pathological topical and systemic reactions in Ps, where local skin-resident cells derived from peripheral blood and skin-infiltrating cells originating from the circulatory system, in particular T lymphocytes (T cells), are key actors. The interplay between molecular components of T cell signalling transduction and their involvement in cellular cascades (i.e. throughout Ca^2+^/CaN/NFAT, MAPK/JNK, PI3K/Akt/mTOR, JAK/STAT pathways) has been of concern in the last few years; this is still less characterised than expected, even though some evidence has accumulated to date identifying them as potential objects in the management of Ps. Innovative therapeutic strategies for the use of compounds such as synthetic Small Molecule Drugs (SMDs) and their various combinations proved to be promising tools for the treatment of Ps *via* incomplete blocking, also known as modulation of disease-associated molecular tracks. Despite recent drug development having mainly centred on biological therapies for Ps, yet displaying serious limitations, SMDs acting on specific pathway factor isoforms or single effectors within T cell, could represent a valid innovation in real-world treatment patterns in patients with Ps. Of note, due to the intricate crosstalk between intracellular pathways, the use of selective agents targeting proper tracks is, in our opinion, a challenge for modern science regarding the prevention of disease at its onset and also in the prediction of patient response to Ps treatment.

## 1 Introduction

Autoimmune diseases are reaching epidemic levels, in part, because there are so many of them. Current medicine lists between 80 and 100 different types of autoimmune disorders or diseases. Some of the more common ones include Psoriatic (Ps) disease, a non-communicable skin and/or joint condition, currently regarded as an immune-mediated inflammatory disease (IMID) (1–4). Various molecular and cellular pathways are implicated in Ps development and progression, as well as in the pathogenic mechanisms associated with an inflammation in this hyperimmune condition. Our understanding of this complex network and its tight regulation, however, turns out to be still in its infancy. Such a situation, while frustrating, offers an opportunity for further progress in the development of therapies thanks to the potential increase in our knowledge that is taking place now. It actually allows us to refine the current treatment options as well as define novel ones, focused not only on stopping attacks in progress and managing symptoms of Ps, but also on targeting the roots of disease processes. This is trying to be accomplished by using therapeutic compounds, such as Small Molecule Drugs (SMDs), developed for innovative strategies targeting any portion of a molecule, regardless of the target’s cellular location, in order to depress the immune system and reduce inflammation, alleviating other symptoms at the same time (5–7). Recent advances in understanding the pathogenesis of the Ps have already led to the development of a number of SMDs that have a low molecular weight, making them penetrate cells easily and affecting molecular pathways by targeting important cellular entities. They have advantages like oral routes of administration, decreasing healthcare costs and fewer immunological adverse events compared to biologics (6). Besides, we would like to point out the fact that SMDs can be developed not only from leads derived from rational drug design, but also isolated from natural resources, leading to the innovation of more desirable treatments with minimum side effects possible.

In Ps, both skin-associated cells and those recruited from the circulatory system, belonging to the acquired and innate immune systems, especially T lymphocytes (T cells), are involved in complex feedback mechanisms of the disease pathophysiology (8–13). The discovery that topical treatments for skin symptoms alter the T cells’ phenotype has drawn a special attention to the molecular pathways modulated in immune cells under Ps (14). Except for the intracellular cross-talk, however, the interplay between the molecules involved in different signalling tracks (i.e. throughout calcium (Ca^2+^)/calcineurin (CaN)/nuclear factor of activated T-cell (NFAT), mitogen-activated protein kinase (MAPK)/c-Jun N-terminal kinase (JNK), phosphoinositide-3-kinase (PI3K)/protein kinase B (Akt)/mammalian target of rapamycin (mTOR), Janus kinase (JAK)/signal transducer and activator of transcription (STAT) pathways) in T cells is of concern in the last few years, identifying them as potential targets in the management of Ps (7). At the same time, it became obvious that larger and further studies are desirable to fully explore signalling cascades involved in the pathogenesis of the disease, especially to accomplish knowledge gaps regarding pathological mechanisms within psoriatic T lymphocytes. The creation of Ps disease atlas showing a road map of intracellular molecular pathways and their regulation network supported by a library of therapeutic entities would be a highly respected and lucrative approach in the medical community as per our vision. A rather wide point of view must be mapped by connecting each piece of information, which consequently can help disease navigate and curing. Furthermore, the “new use of old medicine” would also refer to the development of new indications or new uses of drugs that were previously marketed for other purposes. Such innovative approach to research fits perfectly into the “me-too” strategy era (15), allowing to find the most appropriate molecule dedicated to the most effective disease regulation mode. This mini-review provides a succinct summary on the current progress in research of molecular treatment trajectories within psoriatic T lymphocytes and serves to encapsulate the recent literature regarding this aspect.

## 2 Insights into the determinants contributing to the expression of Ps

Almost all types of skin-resident cells are involved in the pathogenesis of Ps, most notably non-immune epidermal keratinocytes (KCs) and accompanying dermal fibroblasts, as well as skin-resident immune cells, such as dermal γδ T cells, macrophages, Langerhans cells (LCs), natural killer cells (NKs), dendritic cells (DCs), mast cells (MCs), group 3 innate lymphoid cells (ILC3s) and vascular endothelial cells. The immune response also recruits cells from the circulatory system, such as mobile T cells, B cells, granulocytes, monocytes, and MCs (16–21). The disease is mediated not only by the irregular function of cells, but also by the presence of molecules of both the innate and adaptive immune systems, mainly cytokines which are, under normal conditions, involved in constitutive or inducible pathways, but are significantly amplified in Ps.

Studies have shown that the dynamic interplay between cells together with the molecular environment is dysregulated in Ps, leading to a persistent inflammatory process, modulated mainly by the activation of T lymphocytes and pro-inflammatory cytokines (22–24). It has been a long-standing discussion as to whether the primary process in Ps involves epidermal overgrowth of KCs with secondary immune activation mediated by T cells or *vice versa*, informed by a plethora of studies on potential drugs used for Ps treatment (25). The identification of early triggers, i.e., autoantigens and the key role played by immune cells, mainly T helper (Th) cells, in the front line in the early pathogenic steps of Ps is already well-documented (9, 26, 27). The discovery of the common subunit of interleukin (IL)-12 and IL-23 has pointed to the fact that autoimmune diseases, including Ps, are not only induced by Th1/Th2 dominancy but also by IL-23 responsive Th cells (28). This lineage, identified as Th17 cells, also produces cytokines such as IL-17A, IL-17F, IL-21 and IL-22 (29, 30); these cells have come to the forefront of the current model of Ps, as an IL-17 blockade can significantly reverse molecular and clinical disease features (3). Also, IL-21, highly expressed in the lesional skin and peripheral blood cells of Ps patients, may promote psoriatic inflammation by inducing an imbalance in Th17 and Treg cell populations. Treg cells are a subset of T cells that suppress the inflammation induced by other T cells, such as Th17 (31). IL-21 promotes CD4-positive (CD4^+^) T cells proliferation, Th17 cell differentiation and the inhibition of Treg cell differentiation by the upregulation of RAR-related orphan receptor γt (RORγt) expression and the downregulation of Foxp3 expression, which results in increased expression and the secretion of IL-17A and IL-22 (32).

**Figure 1:**
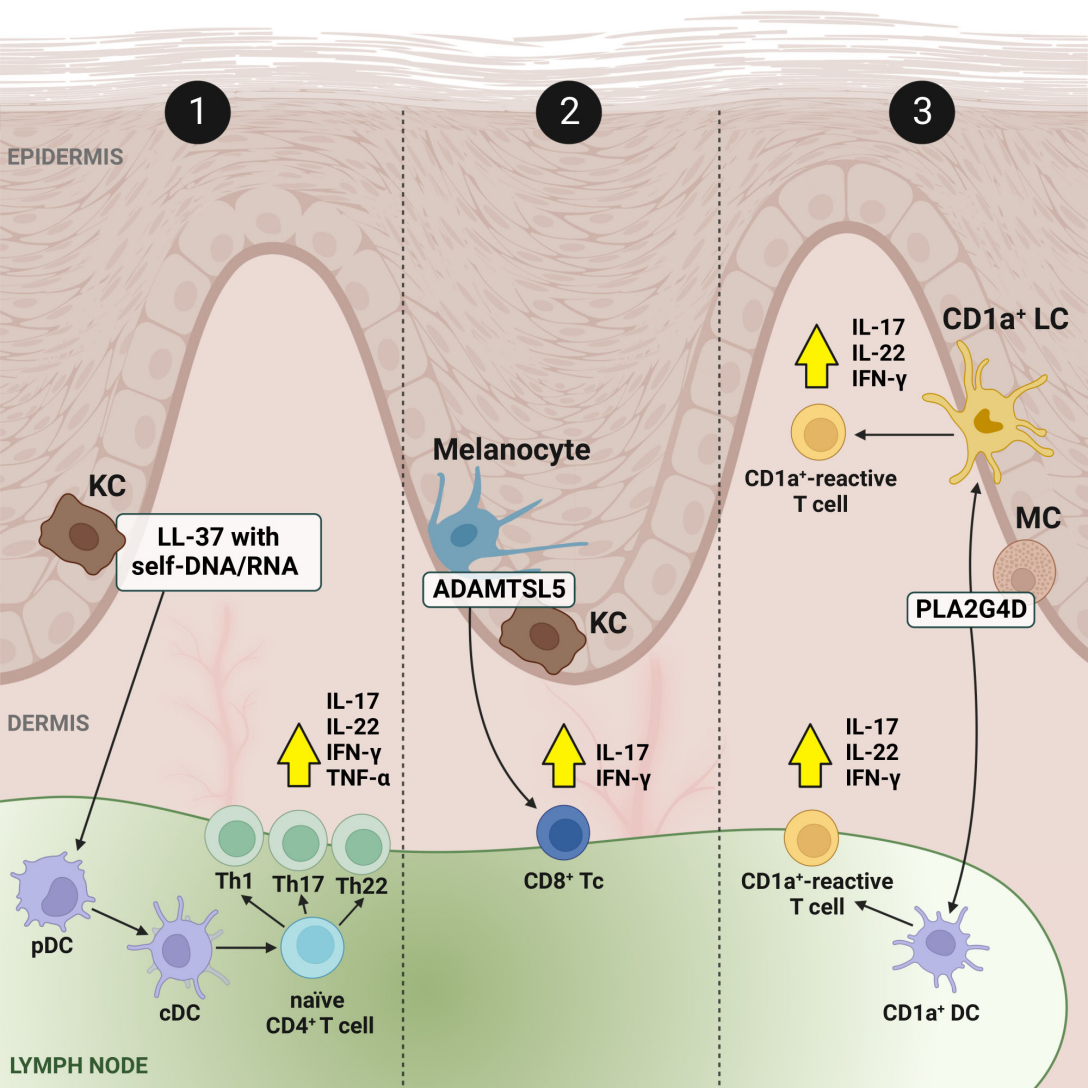
Concepts and present facts of the immune-mediated inflammation circuit in Ps. Feedback signalling loops connect output signals to their inputs in relation to local skin-resident and skin-infiltrating immune cells and cover three conceptual models. In model no. 1: in response to triggers, KCs release autoantigenes, among others cathelicidine LL-37 in a form of complexes with self-DNA/RNA (LL-37/DNA and LL-37/RNA), which bind to pDCs and activate them, and consequently activate cDCs. Stimulated cDCs, acting as APCs promote the expansion of autoreactive T cells, and differentiation from naïve CD4^+^ T cells to Th1, Th17 and Th22. It results in the production of inflammatory cytokines (production of the most important, i.e., IL-17, IL-22, IFN-γ and TNF-α, is depicted by yellow arrows in a black frame), but also chemokines and AMPs which further activate immune cells, enabling a positive feedback loop between KCs and T cells that ensure the perpetuation of Ps inflammation. In the other two models (no. 2 and no. 3), autoantigens, ADAMTSL5 in melanocytes and KCs and PLA2G4D in MCs, correspondingly work as triggers. The first is a melanocytic protein (i.e., ADAMTSL5 in melanocytes) which has been described to be an autoantigen to epidermal autoreactive CD8^+^ Tc cells expressing IL-17A and IFN-γ in Ps patients with the risk gene HLA-C*06:02.19, while the second represents a cytoplasmic phospholipase (i.e., PLA2G4D in MCs) producing non-peptide neolipid autoantigens expressed on CD1a^+^ DCs, such as LCs, which, upon PLA2G4D presentation, activate CD1a^+^-reactive T cells secreting IL-17, IL-22 and IFN-γ. Created using BioRender.com. ADAMTSL5, a disintegrin-like and metalloprotease domain containing thrombospondin type 1 motif-like 5; AMP, antimicrobial peptide; CD1a^+^, cluster of differentiation 1a-positive; CD4^+^, CD4-positive; CD8^+^, CD8-positive; cDC, conventional dendritic cell; LL-37, cathelicidin; DC, dendric cell; IFN-γ, interferon γ; IL, interleukin; KC, keratinocyte; LC, Langerhans cell; MC, mast cell; pDC, plasmacytoid dendritic cell; PLA2G4D, phospholipase A2 group IVD; Ps, psoriasis; Th, T helper; TNF-α, tumor necrosis factor α.

To date, there are three concepts regarding the initiation of Ps (**Figure 1**). The first concept assumes two hypotheses: either with DCs acting as antigen-presenting cells (APCs) and simultaneously stimulating T cells to produce cytokines for the subsequent activation of KCs, or with KCs as direct APCs leading to T cell activation; the second concept concerns ADAMTS-like protein 5 (ADAMTSL5) autoantigens in melanocytes and KCs as triggers activating T cells; and the third concept refers to phospholipase A2 group IVD (PLA2G4D) autoantigens expressed on skin CD1a^+^ DCs (CD1a^+^ LCs), which activate CD1a^+^-reactive T cells upon presentation (8). It has to be mentioned that the conceptual models of the initiation phase of Ps pathogenesis have been shown only *in vitro* and they are considered to be assumptions. Whether they can be transformed to clinical relevancy remains uncertain (10, 33). Ps continues to be classically perceived as a T cell-mediated inflammatory disorder. Although it is now well-recognised that T lymphocytes do not function in exclusivity to induce Ps alterations, these cells are identified as potential targets in the management of the disease (34–39). At the same time, it should be remembered that skin-associated cells also play an important functional role during Ps progression. For this reason, literature reports on the pathogenesis of Ps swing the pendulum between KC and immune cells, undoubtedly pointing to them as the main players. Nevertheless, in this mini-review we debate the potential regulation of dysfunctional key immune cells of the T cell family.

## 3 T lymphocyte fate and commitment of immune- and inflammation-associated molecular patterns of these cells in Ps

A dynamic interaction between skin-resident and circulating immune T cells is visible in Ps. Most T lymphocytes are αβ T cells; however, less common than αβ T cells, but significantly enriched in epithelial sites such as the skin, are γδ T lymphocytes (37), with prevalent contribution in IL-17 production both in the imiquimod (IMQ)-induced Ps-like skin inflammation model and human Ps lesions (40–44), rarely found in healthy people. Although γδ T cells account for only a small fraction of the total T cell pool, emerging evidence suggests that inappropriately activated γδ T cells may play a role in the pathogenesis of Ps (37, 45–49). γδ T cells, considered to bear a memory phenotype are suggested to have critical role in disease recurrence as new Ps lesions often appear in the place of previous ones (49–51). To support this thesis, recent studies revealed that trafficking pattern changes of pathogenic γδ T17 cells play a critical role in skin inflammation recrudescence in Ps-like dermatitis (52). Furthermore, inhibiting the inflammatory response mediated by IL-17-secreting γδ T cells contributes to the attenuation of psoriasiform inflammation (53).

**Figure 2:**
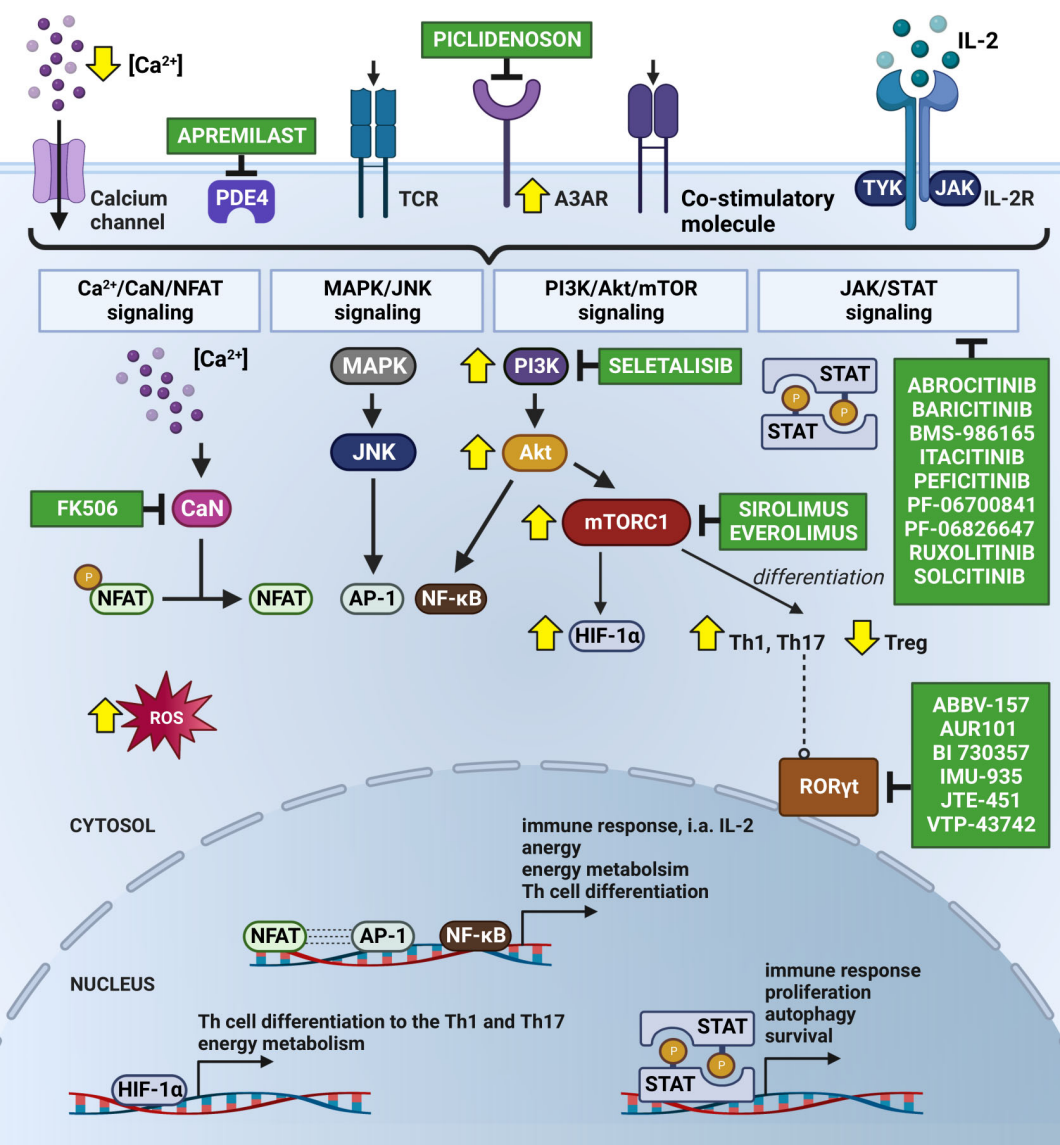
Subjugation of T cells’ Ps immune-mediated inflammatory cascade by Small Molecule Drugs (SMDs). The Ps inflammatory episode begins with an influx of Ca^2+^ into the cytosol followed by the activation of immunoreceptors on the T cell surface, i.e., by antigen binding to the TCR. As a consequence, the nuclear translocation of NFAT occurs upon CaN-dependent dephosphorylation stimulated by calcium-modulated protein CaM. This results in the formation of a transcriptional complex with AP-1. The NFAT : AP-1 complex regulates the expression of immune response genes, including those of secretory cytokines, i.e., IL-2. FK506 has been known to prevent the translocation of NFAT into the nucleus due to its CaN-dependent dephosphorylation inhibition. Seletalisib, known as a small-molecule selective PI3kδ inhibitor, showed effects on size and the appearance of Ps lesions, together with a reduction in T cells and neutrophils (59). TCR activation and co-stimulatory signals force the continuous activation of NFAT, AP-1 and NF-κB throughout the Ca^2+^/CaN/NFAT, MAPK/JNK, PI3K/Akt/mTOR and JAK/STAT pathways, leading to the increased transcription of anti-apoptotic and survival genes. Secreted IL-2 autocrine affects T cells, stimulating their proliferation and differentiation and autophagy, likely mediated by T-cell activation *via* JAK 1/2/3 and TYK2 and STAT proteins (STAT1-STAT6). Abrocitinib, Baricitinib, BMS-986165 (currently known as Deucravacitinib), Itacitinib, Peficitinib, PF-06700841, PF-06826647, Ruxolitinib and Solcitinib are among the small molecule drugs with the role of JAK/STAT inhibitors undergoing clinical trials for Ps. Co-stimulation is important in intracellular cAMP degradation by PDE4. In T cells, PDE4 plays a critical role in signal transduction through recruitment to the cell surface upon concomitant stimulation, where it reduces local cAMP levels. PDE4 inhibition (e.g., by Apremilast) showed exquisite effects on the TCR-induced activation of T cells, manifesting in a reduction in the release of cytokines and chemokines from Th cells (60, 61). Moreover, cAMP modulation is mechanism of another substantial Ps inflammatory pathway with involvement of A3AR, highly expressed on PBMCs in Ps patients. Piclidenoson, an A3AR agonist, downregulates in turn the expression levels of PI3K/Akt, resulting in the depletion of NF-κB, inhibition of TNF-α and apoptosis of inflammatory cells. T-cell activation also supports the production of ROS, involved in NFAT, AP-1 and NF-κB activation and translocation to the nucleus, but also in apoptosis and hyporesponsiveness. Therefore, the consumption of natural compounds with antioxidant properties by Ps patients is highly recommended. Signalling through mTORC1 specifically regulates the T cell activation and differentiation (promotes differentiation into Th1 and Th17 subpopulations and decrease in Treg), autophagy, lysosomal metabolism and metabolic programs in T lymphocytes, which confirms the existence of common regulators of these processes. The suppression of mTORC1 with rapamycin (Sirolimus) favours the generation of Treg cells *in vitro*, even in the presence of Th17-polarising cytokines (62). In addition, a combination of mTORC1 and CaN/NFAT inhibitors, Everolimus and FK506 is validated for recalcitrant Ps treatment. In turn, the activation of mTORC1 during inflammation increases the expression of HIF-1α, a key mediator of the switch from catabolic to anabolic metabolism, which is highly expressed in Ps skin. Additionally, HIF-1α regulates the balance between Foxp3^+^ Treg and Th17 cells, i.e., it weakens Treg lymphocyte development by binding to Foxp3 and directing it to degrade, thereby enhancing Th17 cells by the transcriptional activation of RORγt. Thus, the inhibition of RORγt *via* small molecules (i.e., ABBV-157, AUR101, BI 730357, IMU-935, JTE-451 and VTP-43742) is advised for the treatment of Ps. The changes in Ps T lymphocytes are indicated by yellow arrows. Created using BioRender.com. A3AR, A3 adenosine receptors; Akt, protein kinase B; AP-1, activator protein 1; Ca^2+^, calcium ion; CaM, calmodulin; cAMP, cyclic adenosine monophosphate; CaN, calcineurin; CD28, cluster of differentiation 28; Foxp3, forkhead box P3; HIF-1α, hypoxia inducible factor 1α; IL-2, interleukin 2; IL-2R, interleukin 2 receptor; JAK, Janus kinase; JNK, c-Jun N-terminal kinase; MAPK, mitogen-activated protein kinase; mTOR, mammalian target of rapamycin; mTORC1, mammalian target of rapamycin complex 1; NFAT, nuclear factor of activated T-cell; NF-κB, nuclear factor κB; PBMC, peripheral blood mononuclear cells; PDE4, phosphodiesterase 4; PI3K, phosphoinositide-3-kinase; Ps, psoriasis; RORγt, RAR-related orphan receptor γt; ROS, reactive oxygen species; STAT, signal transducer and activator of transcription; TCR, T cell receptor; Th, T helper cells; Th1, T helper cells 1; Th17, T helper cells 17; TNF-α, tumor necrosis factor α; Treg, regulatory T cell; TYK2, tyrosine kinase 2.

In chronic inflammatory diseases such as Ps, NFAT, activator protein 1 (AP-1) and nuclear factor κB (NF-κB) are essential transcription factors that regulate T cell responses. Their induction leads to the expression of genes that control the growth, expansion, and function of lymphocytes (54, 55). The activation of naïve T cells requires at least two stimuli and optimally a third one, i.e., (*i*) the binding of the antigen on the surface of the T cell receptor (TCR), (*ii*) stimuli derived from co-stimulatory molecules and (*iii*) signals from the common receptor γ-chain, activated by cytokines such as IL-2, IL-4, IL-7 and IL-15 (56–58) (**Figure 2**), which in turn are described in detail in the subsequent subsections devoted to each of them.

### 3.1 Activation of T lymphocytes by TCR stimulation in relation to inflammatory transcriptional program in Ps

Under physiological conditions, TCR stimulation activates phospholipase C-γ1 (PLC-γ1), which catalyses the hydrolysis of phosphatidylinositol 4,5-bisphosphate (PIP2) to inositol 1,4,5-triphosphate (IP3) and diacylglycerol (DAG), thus promoting Ca^2+^ flux. In Ps, shortly after the increase in cellular Ca^2+^ levels, the calcium-modulated protein calmodulin (CaM) transiently triggers 5’AMP-activated protein kinase (AMPK), which, among others, supports the production of reactive oxygen species (ROS) and ATP by mitochondria. ROS and ATP are involved in the activation of NF-κB and AP-1, as well as in the enhancement of NFAT stimulation. However, excess amounts of ROS can result in mutation and cell damage due to their ability to damage DNA and other subcellular structures. Patients with Ps show a redundancy in ROS production and impaired antioxidant protection in both skin cells and blood cells, including lymphocytes (63–65). A positive correlation was observed between the markers of oxidative stress in the serum and blood cells of Ps patients and the disease severity index (66). For this reason, broad spectrum natural compounds with antioxidant properties (e.g., *Aloe vera* L., Bergamot essential oil, Quercetin, Baicalein, Curcumin and Resveratrol) are routinely included in the daily diet of most of Ps patients, despite there being limited information about the possible mechanisms of action of such treatment (67).

CaM also stimulates calcineurin (CaN), calcium and calmodulin-dependent serine/threonine protein phosphatase, which dephosphorylates NFAT; in this condition, NFAT is translocated into the nucleus (55, 57). At the genomic level, the decision between T cell activation and anergy depends on whether NFAT, upon its stimulus-induced nuclear translocation, forms a transcription factor complex with AP-1, a dimer composed of proteins belonging to Jun, Fos and activating transcription factor (ATF) protein families (68). The transcriptional program induced by NFAT:AP1, which includes IL-2, IL-4, IL-5, IL-13 and IFN-γ expression, initiates a productive immune response, whereas genes induced by NFAT lead only to T cell tolerance. NFAT also plays a key role in the regulation of CD4^+^ Th cell differentiation, mediated by the gene expression control of the transcription factors T-bet (Th1), Gata3 (Th2), RORγt (Th17), FOXO4 (Th22) and Foxp3 (Treg) (29, 69–74). As NFAT is regulated in a highly dynamic manner by Ca^2+^, a decrease in intracellular Ca^2+^ levels results in the almost immediate phosphorylation and export of NFAT from the nucleus. As a consequence, NFAT-dependent gene transcription is only poorly activated in response to a single pulse of high intracellular Ca^2+^ but requires the prolonged increase of Ca^2+^ (75, 76). Of note, hypocalcaemia was found to be a risk factor of Ps, as decreased levels in the serum and disturbances in the metabolism of Ca^2+^ in Ps patients were visible (77, 78).

### 3.2 Activation of T lymphocytes by co-stimulatory molecules in relation to cellular signalling cascades in Ps

Co-stimulation of T lymphocytes, provided by the interaction between co-stimulatory molecules, is necessary for the activation and translocation of AP-1 to the nucleus, i.e., for the formation of the NFAT:AP-1 complex (76). Special attention has been paid at this time to the PI3K/Akt and MAPK/JNK molecular pathways, both of which are involved in AP-1 and NF-κB activation and translocation to the nucleus (79, 80). Interestingly, PI3K is relevant for IMQ-induced skin inflammation, as well as IL-17 secretion by CD4^+^ γδ T cells in a Ps mouse model (81). Moreover, the blocking of PI3K inhibits the production of IFN-γ, IL-4, IL-6, IL-13, IL-17A, IL-22 and GM-CSF by human memory and γδ T cells (81). PI3K and Akt are also upregulated in the peripheral blood mononuclear cells (PBMCs) of Ps patients (82). Seletalisib, a small-molecule selective PI3kδ inhibitor, efficiently reduces the secretion of IFN-γ, IL-17 and TNF-α by Ps T cells *in vitro* (83). Investigation with the oral administration of Seletalisib proved the remission of Ps lesion size and appearance, along with a reduction in skin-resident T cells and neutrophils (84).

In the PI3K/Akt pathway, mTORC1 is also activated (85–87). Mechanisms, or rather how mTORC1 signalling controls cell homeostasis and contributes to inflammatory skin diseases such as Ps, are broadly discussed in the literature (88–90). The analysis of PBMCs from Ps patients has shown an increase in mTORC1 signalling (82). For this reason, mTORC1 inhibitors, like rapamycin (Sirolimus) (62) along with its derivative Everolimus, are used for Ps treatment (91). These drugs reduce T cell activation, prevent cell cycle progression, promote autophagy, induce anergy in naïve T lymphocytes and forward the differentiation into Foxp3^+^ Treg cells (92, 93). Systemic treatment with rapamycin inhibits the secretion of IL-17 and IL-22 by CD4^+^ γδ T cells (40). Rapamycin interacts with FKBP-12 protein and as the rapamycin:FKBP-12 complex binds to FKBP12–rapamycin-binding (FRB) domain, this results inter alia in restricted access to the binding site of mTOR substrates and inhibition of the phosphorylation of some mTORC1 targets. Furthermore, rapamycin in the presence of FKBP-12, inhibits the association of raptor (regulatory associated protein of mTOR) – mTORC1 binding partner, essential for mTORC1 signalling and simultaneously reduced the mTORC1-catalysed phosphorylation of raptor-dependent substrates. As a consequence, the effects of mTORC1 both on cell growth, autophagy and cell cycle progression are being constrained (94–97). Another drug used in Ps, which also binds to the FKBP-12 protein, is Tacrolimus (FK506). The FK506:FKBP-12 complex does not react with mTOR, whereas it inhibits CaN-dependent dephosphorylation of NFAT and prevents the translocation of NFAT into the nucleus (98). A combination of mTORC1 inhibitors Everolimus and Tacrolimus was tested in recalcitrant Ps treatment (99).

The co-stimulation of CD28 and T-cell receptors is important in intracellular cyclic adenosine monophosphate (cAMP) degradation by phosphodiesterase (PDE) 4. PDE4, as one of the dominant PDEs, which are enzymes responsible for the hydrolysis of cAMP, is a key second messenger that controls a network of pro-inflammatory and anti-inflammatory mediators, expressed in immune cells (100). In T cells, PDE4 plays a critical role in signal transduction upon concomitant stimulation, by the reduction of cAMP levels. PDE4 inhibition showed exquisite effects on TCR-induced activation of T cells, manifesting in the reduced release of cytokines and chemokines from Th1, Th2 and Th17 lymphocytes (60, 61). Apremilast (Otezla) is among the small molecules that inhibit PDE4, leading to increased intracellular cytosolic cAMP and the activation of protein kinase A (PKA) (61).

cAMP modulation is a mechanism of another substantial Ps inflammatory pathway with involvement of A3 adenosine receptors (A3AR), highly expressed on PBMC in Ps patients. Piclidenoson (CF101), an A3AR agonist has been found to downregulate the expression levels of PI3K, Akt, IκB kinase (IKK) and the inhibitor of NF-κB (IκB) resulting in the down-regulation of NF-κB, the inhibition of IL-6, IL-12 and TNF-α and the apoptosis of inflammatory cells. In addition, a direct anti-proliferative effect of CF101 towards auto-reactive T cells was observed (101, 102). As A3AR is over-expressed on a set of Ps immune-system cells, the other inflammatory cells are resistant to Piclidenoson treatment (103).

### 3.3 Activation of T lymphocytes by signals from the γ-chain receptor in relation to molecular signal transduction in Ps

In the absence of co-stimulatory signals, AP-1 activation declines and NFAT initiates the anergy transcription program, characterised by a decrease in TCR expression and IL-2 production in response to the antigen (68, 104, 105). It is worth emphasising the role of IL-2 as one of the cytokines produced as a result of T cell activation that is crucial for sustained T cell proliferation and survival. This pathway and others with cytokines that are pivotal for Ps pathogenesis (i.e., IFNs, IL-6, IL-22, IL-23) are mediated by Janus kinase (JAK) 1/2/3 and tyrosine kinase 2 (TYK2), as well as the signal transducer and activator of transcription (STAT) proteins (STAT1-STAT6) (106, 107). The inhibition of each subtype of JAK family members by small molecules can interfere in molecular pathways and have critical significance; on the other hand, it results in the signalling block of many cytokines, which makes them less selective than monoclonal antibodies. Abrocitinib, Baricitinib, BMS-986165 (currently known as Deucravacitinib), Itacitinib, Peficitinib, PF-06700841, PF-06826647, Ruxolitinib and Solcitinib are among the small molecule drugs with the role of JAK/STAT inhibitors undergoing clinical trials for Ps (6, 107, 108). STAT1 and STAT3 are involved in the differentiation and effector function of Th1 and Th17 (6, 109) and may therefore serve as therapeutic targets. STAT1 deficiency restrains the IL-27-mediated suppression of IL-17 and IL-22 (110, 111), while STAT3 is requisite for IL-21-induced IL-22 expression in CD4^+^ T cells (112). Aryl hydrocarbon receptor (AhR) participates in the differentiation of Th17 cells by regulating STAT1 and STAT3 activation (113).

## 4 T cell metabolism program upon activation with special reference to Ps cellular signalling

Decisions about the fate of T lymphocytes are shaped by cytokines and growth factors and are related to nutrient-dependent metabolism. Activation of mTORC1 during inflammation increases the expression of hypoxia-inducible factor 1α (HIF-1α), which, together with MYC, up-regulates glucose and amino acid metabolism (114). HIF-1α is a key mediator of the switch from catabolic to anabolic metabolism as it blocks oxidative phosphorylation (OXPHOS) under hypoxic conditions. Fully activated T cells quickly change their metabolic program from catabolic OXPHOS to the anabolic processes of glycolysis and anaplerotic glutaminolysis. One of glycolytic intermediates, phosphoenolpyruvate (PEP), can regulate the Ca^2+^/CaM/CaN signalling pathway, depending on glucose availability. PEP inhibits the sarcoplasmic reticulum Ca^2+^/ATPase pump (SERCA). Consequently, the level of Ca^2+^ in the cytosol is increased, which supports rapid NFAT translocation to the nucleus (55, 115).

NFAT also regulates the expression of HIF-1α and other metabolic regulators such as interferon regulatory factor 4 (IRF4), RORγt and potentially MYC. Interestingly, HIF-1α is highly expressed in Ps skin and statistically correlates with increased levels of IL-6 in Ps serum (116, 117). Additionally, HIF-1α regulates the balance between Foxp3^+^ Treg and Th17 cells, i.e., it weakens Treg lymphocyte development by binding to Foxp3 and directing it to degrade, thereby enhancing Th17 cells by the transcriptional activation of the RORγt. RORγt is a master transcriptional factor of Th17 cells (118), so the inhibition of RORγt is considered to be a promising strategy for the treatment of autoimmune disease (119). Several small molecule inhibitors of RORγt (i.e., ABBV-157, AUR101, BI 730357, IMU-935, JTE-451, VTP-43742) are being investigated in Ps clinical trials (120–122).

## 5 Remarks and future perspectives

It is becoming more evident that specific interactions of inter- and intracellular players within the immune system result in Ps and, furthermore, that their interactome in relation to subcellular signalling machinery orchestrates the choreography of the disease. As estimated by us, SMDs represents a breakthrough Ps treatment approach that does not target any single mediator, as in the case of biologics, but rather focuses on restoring a balance of pro-inflammatory and anti-inflammatory signals. In future studies, it will be quite intriguing and desirable to define the interplay more precisely between the cellular factors and metabolic regulators of Ps-affected signal transduction pathways in order to update perspectives regarding the SMD treatment approach interrupting the pathological cascade earlier in the response or further upstream and returning pro-inflammatory and anti-inflammatory signalling to a homeostatic balance. We believe that this will allow clinicians to institute targeted and personalised medicine, leading to the maximisation of efficacy and minimisation of toxicity and allowing us to overcome the biggest challenge we face in achieving disease prevention at its onset as well as in the prediction of patients’ response to Ps treatment. In addition, we have a strong conviction that although the drug discovery still relies primarily on cell and animal models that mostly use phenotypic screenings, further exploration needs the implementation of computational biology, bioinformatics, molecular biology, and chemical biology in order to identify the appropriate molecular treatment trajectories within the objects that are key actors in the disease, and which in Ps are T lymphocytes.

## Author contributions

Original draft preparation, review, and editing: MK, MG-C, MM; supervision, funding acquisition: MG-C, MM. All authors have read and agreed to the published version of the manuscript.
